# Thymus activity measured by T-cell receptor excision circles in patients with different severities of respiratory syncytial virus infection

**DOI:** 10.1186/s12879-016-2148-0

**Published:** 2017-01-05

**Authors:** Kiran Aftab Gul, Tonje Sonerud, Hans O. Fjærli, Britt Nakstad, Tore Gunnar Abrahamsen, Christopher S. Inchley

**Affiliations:** 1 Department of Pediatric Research, Women and Children’s Division, Oslo University Hospital Rikshospitalet, Oslo, Norway; 2 Department of National Newborn Screening, Women and Children’s Division, Oslo University Hospital Rikshospitalet, Oslo, Norway; 3 Department of Pediatric and Adolescent Medicine, Akershus University Hospital, Lørenskog, Norway; 4 Department of Clinical Molecular Biology and Laboratory Sciences (EpiGen), University of Oslo, Akershus University Hospital, Lørenskog, Norway; 5 Institute of Clinical Medicine, University of Oslo, Oslo, Norway; 6 Clinic of Pediatric and Adolescent Medicine, Women and Children’s Division, Oslo University Hospital Rikshospitalet, Oslo, Norway

**Keywords:** Respiratory syncytial virus, Thymus, T-cell receptor excision circles, Children, Bronchiolitis

## Abstract

**Background:**

*Respiratory syncytial virus* (RSV) infection is an important cause of hospitalization in previously healthy infants. Immunological mechanisms predisposing infants to severe disease are poorly understood. Early biomarkers for disease severity may assist clinical decisions. We investigated T-cell receptor excision circles (TREC), episomal DNA made during thymic T-cell receptor rearrangement, and a marker for thymus activity, both during disease and in neonatal screening cards as a risk factor for RSV disease severity.

**Methods:**

One hundred thirteen patients hospitalized with RSV infection <12 months of age, grouped by disease severity, were available for this investigation, in which we conducted both a prospective and a case-control study. The prospective study included 47 RSV positive infants (mild *n* = 13, moderate *n* = 10, severe *n* = 24). TREC counts were determined by PCR of DNA extracted from EDTA-blood collected on hospitalization, and corrected for lymphocytes using ANCOVA. The case-control study included 85 newborns who later in infancy became RSV positive (mild *n* = 32, moderate *n* = 24, severe *n* = 29) and 47 newborns who never developed RSV disease as healthy controls included from health centres in the same catchment area. TRECs were measured using DNA extracted from dry blood spots from stored neonatal screening cards, followed by PCR. Student’s *T*-test compared patients with controls, ANOVA compared disease severity groups.

**Results:**

During RSV infection patients in the severe disease group had significantly lower (*p* = 0.017) TREC/200 μL blood compared to the other two disease groups, after correction for lymphocyte count. Newborn TREC levels, were significantly higher in RSV patients compared to controls (*p* < 0.0001). No significant differences in TREC copies at birth were found between disease severities.

**Conclusion:**

During acute RSV infection a lower number of TREC is found in the severe disease group. TREC has potential as an immunological marker for severe RSV infection. Higher neonatal TREC counts indicate that infants later presenting with severe RSV do not have reduced thymic activity at birth and probably no congenital T-cell defect.

## Background

*Respiratory syncytial virus* (RSV) infection occurs in annual winter epidemics. Almost all children are exposed before two years of age [1, 2]. Clinical conditions include mild upper respiratory tract infection, bronchiolitis, pneumonia and respiratory failure. 22–31/1000 infants under one year of age [2, 3] are hospitalized and may require supportive treatment including oxygen, fluids, continuous positive airway pressure (CPAP) and in some severe cases mechanical ventilation [1].

Innate immune cells responding to RSV activate naïve T-cells [2], which have a critical role in termination of an acute RSV infection. The thymus has important biological roles in the generation of T-cells from precursor bone marrow derived cells. T-cell receptor excision circles (TRECs) are circular pieces of DNA generated during the rearrangement of variable V, D, and J segments of T-cell receptor (TCR) genes in the thymus [4, 5]. TREC is an accurate measure of thymic function [6] because it arises in the late phase of thymocyte maturation [4, 5]. It can be used to estimate thymic activity in peripheral blood because intrathymic and peripheral TREC values are correlated [7]. In the case of T-cell lymphopenia very low or undetectable TREC levels will be found in peripheral blood, this reflects the lack of T-cells produced by the thymus [4]. TRECs do not replicate during cell division, and are therefore more abundant in naïve T-cells that have recently emigrated from the thymus. Over 70% of rearranged α/β T-cell receptors form a circular DNA TREC [4]. Newborns have high TREC levels that decline with increasing age together with a peripheral T-cell expansion [8].

In the first part of this study we investigated thymus activity in RSV disease using EDTA blood samples collected during a prospective study of RSV bronchiolitis. The results indicated low TREC counts in the severe disease group. Furthermore, to determine if these patients had low thymus activity (TRECs) already from birth, we quantified TRECs in dried blood spot (DBS) of newborn screening (NBS) filter cards and compared them to controls. Other thymic markers such as TCR delta chain (TCRD) and the amount of regulatory T cells (Tregs) are also well correlated with thymic capacity [9]; we decided to use TREC because the method has been established in our laboratory as a screening tool for patients with severe combined immunodeficiency.

The aim of this study was to investigate thymus activity in patients with RSV infection, both at birth in those who later developed RSV disease and during disease, and the impact of thymus activity on disease severity.

## Methods

During the RSV season in Akershus and North Oslo, Norway, from January – March 2011, infants < 12 months of age with respiratory tract infection examined at the paediatric emergency unit, Akershus University Hospital were considered for inclusion in a study of nasal mucosa miRNA expression [10]. One hundred and thirteen patients and 102 controls were recruited. Clinical data and blood samples from patients were collected for later analysis. The controls were recruited from the local health centres.

In our prospective study EDTA blood available in 47 of the included patients were used. The patients were grouped into mild, moderate and severe disease based on clinical data. On inclusion, patients were assessed by the treating paediatrician for respiratory distress using the validated Respiratory Distress Assessment Instrument (RDAI) [11] . The RDAI assesses retractions (max 9 points), wheeze (max 8 points) and changes in respiratory rate. A modified RDAI (m-RDAI) was developed to permit a single observation of respiratory rate and is described further in Table 1 [10]. Children receiving oxygen or fluid supplement, continuous positive airways pressure (CPAP) or mechanical ventilation was classified with severe disease. Children not admitted were classified with mild disease. Children admitted but who did not receive oxygen, fluids, CPAP or mechanical ventilation were classified with moderate disease if they had an m-RDAI 9–25, and with mild disease if they had an m-RDAI 0–8. Clinical characteristics and treatments given are provided in Table 2.

**Table 1:** Modified respiratory distress assessment Instrument (m-RDAI)

Points	0	1	2	3	4
Wheeze					
Expiration	No	End-Expiratory	½ of inspiration	¾ of inspiration	Whole expiration
Inspiration	No	Partly	Whole inspiration		
Location	None	≤2 of 4 lung fields	≥ 3 of 4 lung fields		
Retractions					
Suprasternal	No	Mild	Moderate	Significant	
Intercostal	No	Mild	Moderate	Significant	
Subcostal	No	Mild	Moderate	Significant	
Respiratory rate		Upper limit for normal respiratory rate		Points	
Age < 1 month		50/ min		1 point for each increment of 5 breaths/minute above normal, max. 8 points	
Age 1–5 months		40/ min			
Age 6–11 months		30/ min			

Maximum score for wheeze - 8 points; for retractions – 9 points; for respiratory rate – 8 points. Thus RR, retractions and wheeze are equally weighted. Maximum m-RDAI score is 25 points. Adapted from [11]. Age-associated limits for respiratory rate (RR) were set according to the literature and clinical experience. Example - a 3-month old child with a respiratory rate of 62 receives 5 points for Respiratory rate (62–40 = 22; 22/5 = 4.4; round up to 5)

**Table 2:** Clinical characteristics of RSV-positive infants, categorised by disease severity

	Mild		Moderate		Severe		Sig.
Total number of patients	37		25		40		
TREC during RSV infection, number of patients	13		10		24		
TREC in neonatal screening cards, number of patients	32		24		29		
Age when RSV diseased - months, median (IQR)	3	(1–6)	3	(1–5.5)	2	(1–4)	*p* = 0.4^a^
Male gender, number (%)	14	(38%)	14	(56%)	17	(43%)	*p* = 0.4^b^
Weight - grams, mean (SD)	6522	(2277)	6748	(1935)	5974	(2065)	*p* = 0.4^c^
Duration of symptoms at admission - days, median (IQR)	4	(3–5.25)	4	(3.5–5)	4	(3–5)	*p* = 0.9^a^
Admission, number (%)	20	(54%)	25	(100%)	40	(100%)	
Length of stay, median (IQR)	1	(0–1)	1	(1–3)	4	(2.5–5.5)	*p* < 0.0001^d^
Length of stay > 3 days, number (%)	1	(3%)	3	(12%)	24	(62%)	*p* < 0.0001^e^
Respiratory distress							
SpO_2_ - % on admission, mean (SD)	97.4	(2.6)	97.9	(2.3)	94.7	(4.5)	*p* < 0.0001^c^
Respiratory rate /min on admission, mean (SD)	49	(12)	58	(12)	57	(10)	*p* = 0.002^c^
Respiratory rate score (max. 8), median (IQR)	2	(0.75–4.25)	4	(2.5–6)	4	(2–6)	*p* = 0.007^a^
							*p* = 0.4^d^
Retraction score (max. 9), median (IQR)	1	(0–2)	4	(2.25–6)	2	(1–4)	*p* < 0.0001^a^
							*p* = 0.003^d^
Wheeze score (max. 8), median (IQR)	0	(0–3)	5	(4–6)	2	(0–4)	*p* < 0.0001^a^
							*p* = 0.0002^d^
m-RDAI, median (IQR)	6	(2–8)	13	(10.5–16)	8	(6–11)	*p* < 0.0001^a^
							*p* < 0.0001^d^
Treatments							
Fluid supplement, number (%)					16	(40%)	
Intravenous fluids, number (%)					4	(10%)	
Nasogastric fluid, number (%)					14	(35%)	
Oxygen supplement, number (%)					35	(88%)	
CPAP, number (%)					4	(10%)	
Respirator, number (%)					0	(0%)	

Of 113 patients available for the study, 92 were included. Fourtyseven had TREC analysed during acute RSV infection, 85 had TREC analysed in neonatal screening cards. Values presented and statistical tests are performed using the total number of RSV positive children included in this study. Analyses show similar results when including only infants studied in the analysis of TREC during RSV infection, or only infants studied in the analysis of neonatal TREC (details not presented). There were no significant differences in age, weight, gender or duration of symptoms between disease severity groups. The severe disease group had a lower SpO_2_ on admission, but had less respiratory distress than the moderate group, as measured by the m-RDAI. The retraction and wheeze scores in particular contributed to this difference. Length of hospital stay increased with increasing severity. The most common treatment for children with severe disease was oxygen supplementation. Only four children required admission to intensive care for CPAP. None were ventilated

^a^Kruskal-Wallis test

^b^Chi-Square test

^c^One-way ANOVA

^d^Mann-Whitney test, comparing moderate and severe disease subgroups

^e^Fisher’s exact test, comparing moderate and severe disease subgroups

To investigate TREC numbers at birth dried blood on filter cards taken as part of the national neonatal screening program was used. All 113 RSV-positive patients and 102 control infants were invited to participate in a case-control study, 85 patients and 47 controls consented. Letters of invitation, consent forms and a questionnaire were sent autumn 2014. Controls were asked about chronic diseases, if they had ever been admitted to hospital or been positive for RSV. Patients and controls with congenital heart disease, chronic lung disease including treated asthma or bronchopulmonary dysplasia, neurological disease, Down’s syndrome, hypotonia, failure to thrive, born <34 weeks gestation, or with other specific conditions associated with more severe disease were excluded.

### Sample collection and virus detection

Infants with suspected RSV infection underwent deep nasal aspiration. Two ml viral transport medium was then suctioned through the suction tube into the sample collection tubes. A rapid antigen test (Abbott TestPack RSV, Abbott laboratories) [12] and/ or multiplex quantitative PCR (qPCR) (in-house test) at the Department of Microbiology, Akershus University Hospital confirmed RSV infection. Blood was collected in 2 ml EDTA tubes on inclusion and stored at -80 °C. Automated differential white blood cell count was performed. Stored blood samples on filter cards from the National NBS Diagnostic Biobank (Oslo University Hospital, Norway) were collected 3 days after birth and stored between -20° and -25 °C. Previous reports demonstrate no significant difference in TREC values between new and old filter cards [13, 14].

### DNA-extraction

Genomic DNA from 200 μl blood was extracted in 100 μl elution solution using the Blood DNA kit (Omega-Biotek, USA) protocol. DNA concentration was measured using Nanodrop (Spectrophotometer ND-1000, USA). A 3.2 mm punch from neonatal screening filter cards was washed with 150 μl DNA Elution solution Qiagen (S2) at 60 °C whilst shaking at 1000 rpm for 10 min in a thermo shaker (TS-100 Biosan, EU) followed by DNA elution in 100 μl S2 at 99.5 °C with continuous shaking at 1000 rpm for 30 min.

### Quantitative PCR

DNA extracts were analysed by qPCR on an ABI Via7 (Applied Biosystems), as previously described [15]. The DNA extract from dried blood spots (DBS) was investigated using the same protocol, modified by using quanta perfecta mastermix (QuantaBio, Beverly, MA).

A TREC plasmid, generated by Douek [4], was provided by the Medical College of Wisconsin, and used to construct the standard curve ranging from 40 000 copies to 10 copies with TREC plasmid concentrations diluted in dilution solution (Qiagen Generation solution 2 containing tRNA 100 ng/μl). Nanodrop (Spectrophotometer ND-1000) was used to measure TREC plasmid concentration. We used β-actin as housekeeping gene to assure adequate DNA extraction. All analyzed qPCR assays had similar slopes and *R* ^2^ values > 0.99.

### Statistical analysis

IBM SPSS version 22.0 was used for statistical analyses. Clinical characteristics were compared between mild, moderate and severe disease groups using One-way ANOVA, Kruskal-Wallis or Chi-squared tests as appropriate. Length of hospital stay and measures of respiratory distress were compared between moderate and severe disease groups using Mann-Whitney or Fisher’s exact tests. One-way ANOVA was used to compare TREC copies and lymphocyte counts between severity groups during RSV disease, with post-hoc analysis using least significant difference (LSD). Univariate linear regression analysis was used to compare TREC copies with lymphocyte counts. Analysis of covariance (ANCOVA) is a general linear model that allows comparison of a continuous variable between groups whilst adjusting for other continuous variables. ANCOVA was used to adjust for effects of lymphocyte count when comparing TREC counts between disease severity groups. Estimated marginal means and 95% confidence interval for TREC copies by group were extracted from the ANCOVA.

TREC copies at birth were not normally distributed and were log transformed prior to statistical analysis. Student’s *T*-test was used to compare controls and RSV positive patients. One-way ANOVA was used to compare TREC copies between controls and the three severity groups with post-hoc testing using Dunnett’s test, in which each severity group was compared to the control group.

## Results

### TREC in RSV positive children during acute illness

**Fig. 1:**
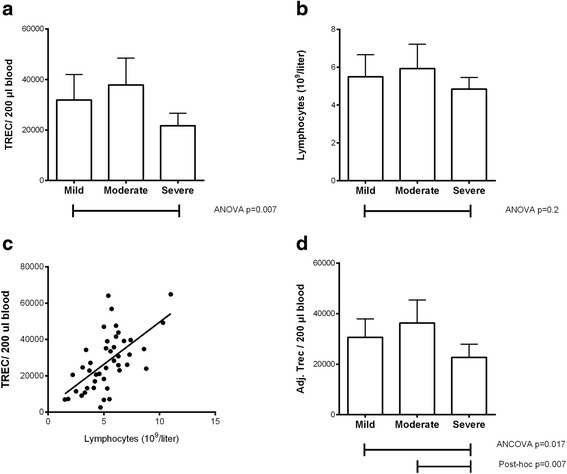
TREC and lymphocyte counts in infants with active Respiratory syncytial virus disease, by disease severity. Note: Panels **a** and **b**: T-cell receptor excision circle (TREC) copy numbers and lymphocyte counts in peripheral blood according to disease severity. TREC counts are significantly lower in the severe disease group. Lymphocyte counts show a similar pattern, but this is not significant. Plots show mean counts with 95% confidence intervals. Panel **c**: Scatterplot of TREC vs. lymphocyte counts. There is a strong positive correlation between TREC counts and lymphocyte counts (*p* = 0.00001; *R* ^2^ = 0.371). Panel **d**: Because TREC counts were strongly correlated to lymphocyte counts, and lymphocyte counts showed a similar pattern to TRECs between groups, TREC counts were adjusted for lymphocytes using ANCOVA. Estimated marginal means with 95% confidence intervals for TREC copies are presented. The difference in TREC count between disease severities remains significant. Post-hoc testing identified the severe disease group as having a lower adjusted TREC count than mild and moderate disease subgroups (*p* = 0.07 and *p* = 0.007, respectively). There was no difference between mild and moderate groups

EDTA blood was available for 47 patients and lymphocyte counts for 64. Significant correlation between TREC copies and disease severity was found (*p* = 0.007). Mean TREC copies in mild disease group was 29 x 10^3^ TREC/200 μl (95% CI 15–43 x 10^3^ TREC/200 μl), moderate group 40 x 10^3^ TREC/200 μl (95% CI 24–57 x 10^3^ TREC/200 μl), and severe disease group 22 x 10^3^ TREC/200 μl (95% CI 10–33 x 10^3^ TREC/200 μl). Reduced TREC copies were identified in the severe disease group compared to mild and moderate (*p* = 0.038 and *p* = 0.003, respectively). Lymphocyte counts showed a similar pattern, but was not statistically significant (*p* = 0.21). Since TREC copies were strongly correlated to lymphocyte count (*p* = 0.00001; *R* ^2^ = 0.371), we wished to exclude lymphocyte count as a confounder. On analysis using ANCOVA, disease severity maintained a significant effect on TREC copies (F = 4.6; *p* = 0.017; partial Et^2^ = 0.189), as did lymphocyte count (F = 19; *p* = 0.00009; partial Eta^2^ = 0.328) and the corrected model h F = 12.5; *p* = 0.000007; Partial Eta^2^ = 0.49. Post-hoc testing in this adjusted model confirmed reduced TREC in the severe disease group compared to mild and moderate groups (*p* = 0.07 and 0.007, respectively). Results are shown in Fig. 1.

### TREC in neonatal DBS filter cards from patients and controls

**Fig. 2:**
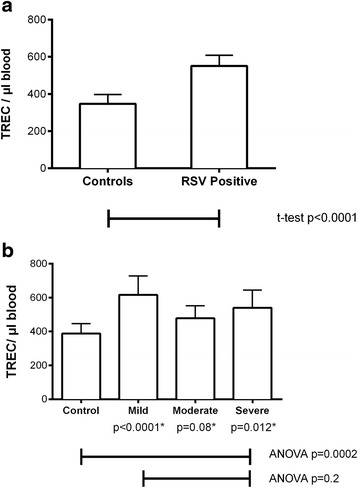
TREC counts in neonatal screening cards in infants who later test positive for Respiratory syncytial virus (RSV), compared to infants who do not. Note: Values shown are mean neonatal TREC copies with 95% confidence interval in neonatal dried blood spot filter cards for 85 RSV positive patients and 47 controls. Panel **a**: infants later testing positive for RSV have significantly higher TREC counts compared to controls. Panel **b**: no significant difference was found between the disease severity subgroups. On post-hoc testing, mild and severe disease subgroups, but not the moderate subgroup, were significantly different to controls. *Post-hoc comparison of each disease subgroup to control, using Dunnett’s test

Eightyfive/113 (75%) RSV-positive (mild *n* = 32, moderate *n* = 24, severe *n* = 29) and 60/102 (59%) controls consented to the neonatal filter card study. Fortyseven (78%) controls were eligible for inclusion. There were significantly higher TREC copies in neonatal filtercards of RSV positive patients (p < 0.0001, Student’s *T*-test); mean TREC copies 551 TREC/μl in RSV positive patients (95% CI 493–609 TREC/μl) compared to 388 TREC/μl in controls (95% CI 329–426 TREC/ μl). There was no significant difference between disease subgroups. Significantly higher TREC copies were found when those who later developed mild and severe disease subgroups were compared to controls (*p* < 0.0001, *p* = 0.012, respectively). Results are shown in Fig. 2.

## Discussion

We found that children assessed at the hospital emergency ward for RSV infection have lower TREC counts in whole blood in cases with severe disease compared to mild and moderate disease. RSV positive patients had higher neonatal TRECs compared to controls, but there were no differences between disease severity groups at birth.

Differentiation between severe disease and moderate disease was based on treatments given, which are objective end-points. Differentiation between mild and moderate groups was based on an assessment of respiratory distress on admission. Follow up assessment of respiratory distress during admission may have identified patients in the mild group who qualified for the moderate group. There may also be some overlap between mild disease cases and controls. Controls did not present to paediatric units in our region and the general practitioner of these patients did not send a nasopharyngeal aspirate for RSV detection. However, almost all children are exposed to RSV by age two years and it is therefore highly likely that controls have also been exposed to RSV, but had a mild course of infection [2].

### RSV positive children during acute illness

We found lower TREC counts in the whole blood of severe RSV patients, but no significant difference in lymphocyte levels. The literature suggests several mechanisms that might explain reduced TREC counts in severe RSV disease: i) reduced thymic activity, ii) increased lymphocyte apoptosis, or iii) redistribution of lymphocytes to other tissues than peripheral blood. Glucocorticoid-mediated thymic suppression is associated with stress [16], which is more likely in severe disease. This concurs with previous studies reporting decreased lymphocytes associated with severe stress in septic patients and after cardiac bypass [17]. Previously published studies report a reduction in absolute lymphocyte counts in children with acute RSV, with most pronounced effects in children who require intensive care [17]. Depletion of T-lymphocyte activity has been associated with mechanical ventilation [18] and a longer duration of oxygen supplementation in infants with RSV infection [19]. The symptoms and signs of RSV disease in our patients varied. Only a few received intensive care and none were ventilated. This patient cohort is therefore representative for the great majority of RSV positive patients presenting to hospital, but not for those with the most severe forms of RSV infection. This may explain the discrepancy between our study and previous reports. A study of peripheral blood during RSV infection did not find differences in T-lymphocyte cytokines between disease severity groups, but lymphocytes had a reduced lymphoproliferative response on stimulation with phytohaemagglutinin [20].

Increased lymphocyte apoptosis in patients with acute RSV infection was suggested in a study by Roe et al. [21] in which expression of the apoptosis-promoting proteins Fas and tumor necrosis factor-related apoptosis-inducing ligand (TRAIL) receptor on CD4^+^ and CD8^+^T-cells during the acute phase was upregulated compared to recovery phase samples [17]. Finally, redistribution of peripheral blood lymphocytes to the lungs during RSV disease was reported [22], and was most pronounced in severe disease. This is in contrast to our study, as lymphocyte levels were not significantly lower in severe disease, which may reflect the paucity of patients requiring mechanical ventilation.

Our study took advantage of samples already available in a biobank and therefore had several limitations. It would have been useful to measure other markers of lymphocyte activity, and to differentiate between T-lymphocytes, including T-lymphocyte subtypes, and B-lymphocytes. In our study, blood samples were taken on presentation to hospital, in many cases before the need for oxygen supplementation arose (median SpO_2_ 94.7% on admission in the severe disease group, see Table 1), and compared to other studies no patients were mechanically ventilated. TREC might therefore be a more sensitive biomarker for disease severity than cytokines, lymphocyte levels or lymphocyte responses. Further studies could investigate TREC’s potential to guide clinical decisions.

### Neonatal DBS filter cards from patients and controls

Evaluation of the immune system prior to infection is particularly interesting, because it may uncover deficiencies that predispose infants to more severe disease. In this study we find higher neonatal TREC counts at birth in patients compared to controls. RSV disease severity is thus not associated with reduced thymic activity at birth, and patients are unlikely to carry a congenital T-cell defect. Increased TREC numbers may nevertheless reflect an immature adaptive immune system.

TREC counts increase on production of TREC positive naive T-cells by the thymus, and decrease on cell death or on dilution during peripheral division of the T-cell pool [4, 23]. High TREC levels in our patients may result from a normal continuous thymic output and reduced peripheral expansion of the naive T-cell pool [17]. We have previously published in a prospective study evidence that IL7R gene is down regulated in cord blood of patients later diagnosed with RSV infection [24]. Our present findings concur with these results. Cytokines, most importantly IL-7, has a crucial role in proliferation and survival of the naive T-cell pool [17]. Reduced IL-7R expression causes lower proliferation of naive T-cells. The overall effect may therefore be that neonates who later present to hospital with RSV have a greater proportion of naïve lymphocytes in their peripheral blood compared to other neonates, and reduced peripheral expansion of lymphocytes carrying RSV-antigen receptors. We propose that reduced peripheral expansion or prolonged naive T-cell survival may delay the adaptive immune response, allowing prolonged viral replication and a greater innate inflammatory response, both factors associated with increased disease severity [25–28].

## Conclusion

In acute RSV disease, we found lower TREC counts in infants with severe disease, suggesting that it has potential as a biomarker for severe disease. We found higher TREC numbers in neonatal DBS filter cards in patients than in controls, suggesting that patients later diagnosed with RSV do not have reduced thymus activity from birth and probably no congenital T-cell defect.

## Abbreviations

ANCOVA: Analysis of covariance
CPAP: Continuous positive airway pressure
DBS: Dried blood spots
DC: Dendritic cells
EDTA: Ethylenediaminetetraacetic acid
LSD: Least significant difference
NBS: Newborn screening
qPCR: quantitative PCR
RDAI: Respiratory distress assessment instrument
RR: Respiratory rate
RSV: Respiratory syncytial virus
TCR: T-cell receptor
TRECs: T-cell receptor excision circles
